# Comorbidities and health-related quality of life among rural older community-dwellers in Vietnam

**DOI:** 10.1371/journal.pone.0321267

**Published:** 2025-04-02

**Authors:** Hai Minh Vu, Hao Thi Tang, Vu Minh Hai (B), Cuong Duy Nguyen, My Ha Nguyen, Hanh Thi Kieu Le, Dat Cong Truong, Hien Xuan Luong

**Affiliations:** 1 Department of Trauma, Thai Binh University of Medicine and Pharmacy, Thai Binh, Vietnam; 2 Faculty of Nursing, Nam Dinh University of Nursing, Nam Dinh, Vietnam; 3 Nursing Department, Thai Binh University of Medicine and Pharmacy, Thai Binh, Vietnam; 4 Department of Intensive Care Unit, Thai Binh University of Medicine and Pharmacy, Thai Binh, Vietnam; 5 Faculty of Public Health, Thai Binh University of Medicine and Pharmacy, Thai Binh, Vietnam; Ospedale San Pietro Fatebenefratelli, ITALY

## Abstract

This study explored the patterns of comorbidities and their impact on health-related quality of life (HRQoL) among elderly individuals living in rural communities in Vietnam. A cross-sectional study was conducted across four communes in Thai Binh province. The demographic characteristics and comorbidities of the participants were evaluated, along with their Euroqol-5 dimensions-5 levels (EQ-5D-5L), using a structured questionnaire supplemented by clinical examinations. A multivariate Tobit regression model was applied to assess the relationship between comorbidities and HRQoL. Results showed that a minority of participants (9.5%) were free of comorbidities. Cataracts were the most common condition (61.0%), followed by osteoarthritis (55.4%), rheumatoid arthritis (46.1%), and dementia (39.0%). The average EQ-5D index was 0.806 (SD =  0.184). Pain/discomfort, difficulties with usual activities, and anxiety/depression contributed most to the reduction in the EQ-5D-5L index. Participants with rheumatoid arthritis (β =  − 0.10; 95% CI =  − 0.13, − 0.07) and postural hypotension (β =  − 0.08; 95% CI =  − 0.14, − 0.02) experienced the greatest decrease in EQ-5D index, followed by those with urinary diseases (β =  − 0.05; 95% CI =  − 0.09, − 0.02) and stroke (β =  − 0.05; 95% CI =  − 0.09, − 0.01). This study highlights the high prevalence of comorbidities among the elderly in rural Vietnam, with arthritis, postural hypotension, urinary diseases, and stroke being most strongly associated with reduced HRQoL. Regular screening and monitoring of comorbidities are vital to identify individuals who would benefit most from healthcare interventions to enhance HRQoL.

## Introduction

For recent decades, the substantial demographic shift towards an ageing population has emerged as a prominent social issue [1,2]. Globally, the population comprised of individuals aged 60 years and older is experiencing a more rapid rate of growth compared to any other age demographic, from one billion in 2020 to more than two billion in 2050 [1]. Simultaneously, the upward trend in life expectancy leads to an escalated individual vulnerability to acquiring one or more illnesses throughout one’s lifetime, culminating in an expanding populace of patients with multiple chronic conditions [1–3].

Chronic diseases and multi-morbidity have been widely recognized as factors strongly correlated with disability, reduced functional capacity, and a decrease in quality of life (QoL) [4]. The presence of comorbidity is linked to a decrease in the rate of recovery and an elevated risk of long-term disability and mortality as supported by the literature [5–9]. Existing literature suggests that the occurrence of multiple health conditions in elderly individuals experiencing falls is observed within a wide spectrum (from 25.8%-84.1%) [10–12]. These negative outcomes are also closely associated with increased utilization of healthcare services and heightened costs within the healthcare sector [13]. Healthcare management in older adults poses a significant challenge due to the presence of comorbidities, necessitating the implementation of comprehensive care plans to effectively regulate and handle these concurrent medical conditions [14,15].

The assessment of health-related quality of life (HRQoL) is a crucial measure in evaluating the effectiveness of health and social care interventions. It commonly involves the collection of patient reports, which provide a subjective evaluation of their health condition. The EQ-5D is a widely used instrument, based on generic preference-based measures, utilized to quantify HRQoL to calculate quality-adjusted life years (QALYs) within the context of economic evaluation [16]. Previous research indicates that the EQ-5D instrument is commonly employed in economic assessments conducted for interventions targeting the elderly population [17,18]. However, additional considerations need to be taken into account when assessing HRQoL among the elderly population, which is due to the potential disparities in physical and mental capacities, educational background, and health comprehension between older individuals and their younger counterparts within the general population [17,20]. Prior research synthesized findings from different studies using the EQ-5D instrument and found that the rate of missing values varied across dimensions, with a range of 0% to 10.7% and the completion rate achieved approximately 90% or higher, suggesting high feasibility in the use of EQ-5D tool when measuring HRQoL in the elderly population [19].

In Vietnam, population aging is becoming an increasingly critical issue, gaining prominence in both political and scientific discussions as the country enters the “golden population” stage [20]. Vietnam is undergoing a rapid demographic shift toward an aged society, with the older population (60 + years) increasing from 12.58 million (12.8%) in 2021 to a projected 28.61 million (24.88%) by 2049 [21,22]. This aging trend presents growing challenges in meeting the healthcare and support needs of older adults. The elderly population, particularly in rural areas, is especially vulnerable due to limited access to healthcare services and a lack of tailored healthcare interventions. The development of health policies and strategic plans for elderly care in Vietnam urgently requires a strong evidence base that takes into account the physiological, environmental, and social factors influencing the QoL of older adults. Comorbidities are a key factor in declining HRQoL among the elderly [23,24], with 75.6% of older patients hospitalized for falls having conditions like hypertension and osteoarthritis [25]. Additionally, 55.5% of community-dwelling older adults experience multimorbidity, defined as two or more chronic diseases [25].

The growing recognition of HRQoL as a vital component in clinical decision-making and policy planning is due to its comprehensive assessment of various dimensions affected by treatment, such as mobility, self-care, physical health, and psychological well-being [26]. Despite the significance of HRQoL, there is a lack of research addressing how comorbidities impact the HRQoL of elderly individuals in rural Vietnam. While some studies have focused on specific subgroups, such as elderly individuals with a history of falls [24], these findings cannot be generalized to the broader elderly population. Therefore, this study aims to address this gap by investigating the patterns of comorbidities and their relationship with HRQoL among elderly individuals residing in rural communities across Vietnam.

## Materials and methods

### Study design

A cross-sectional study was conducted across four rural communes in Thai Binh province from January 1 to December 31, 2022. Individuals were eligible for inclusion if they were 60 years of age or older, had resided in the study area for at least 12 consecutive months, were able to independently comprehend and complete the questionnaire, and willingly consented to participate. Exclusion criteria included individuals with limited ambulatory abilities, those who were absent during the study period, and those who declined participation.

The decision to exclude individuals with limited ambulatory abilities was made because the EQ-5D-5L instrument evaluates health-related quality of life across five dimensions: mobility, self-care, usual activities, pain/discomfort, and anxiety/depression. Including individuals with severe mobility impairments could lead to overrepresentation of mobility-related problems, potentially skewing the overall HRQoL assessment. Additionally, only individuals who could comprehend and complete the questionnaire independently were included to ensure the accuracy of self-reported data. This selection criterion aimed to minimize potential bias due to proxy responses, as third-party interpretations could introduce inconsistencies in subjective HRQoL assessments. However, it is acknowledged that these criteria may limit the generalizability of the findings, particularly among the most vulnerable elderly populations with cognitive decline or severe disabilities.

A multi-stage sampling approach was utilized, beginning with a comprehensive listing of rural communes in Vu Thu district, from which four communes were randomly selected, each with a population exceeding 8,000 individuals, including approximately 800–1,000 elderly residents per commune. The required sample size was determined using the formula for estimating a population proportion with specified absolute precision, with an estimated 55.5% prevalence of comorbidities (p =  0.555) [25], a margin of error (d =  0.018), and a 95% confidence level (Z =  1.96), resulting in a minimum required sample of 2,937 participants. To account for potential non-responses, we increased the sample size by 5%, setting the final requirement at 2,929 participants. With assistance from healthcare professionals at district health facilities, all eligible elderly residents within the selected communes were identified and invited to participate. A total of 3,140 elderly individuals were contacted, and 3,038 voluntarily participated, yielding a response rate of 96.8%, surpassing the required sample size for statistical reliability. This study was approved by the Scientific and Ethical Committee of Thai Binh Province (Code: 2320/QD-UBND) and the Thai Binh University of Medicine and Pharmacy.

### Data measurement

The team collecting data included researchers and medical students from Thai Binh University of Medicine and Pharmacy, as well as health workers from four community health centers. All data collectors underwent intensive training sessions conducted by field specialists to develop strong interview and communication abilities. The participants were familiarized with the purpose and characteristics of the questionnaire to ensure their comprehension and active participation in the study.

The supervisors consisted of the main researchers and the leaders of the community health facilities. They actively participated in a training program alongside the investigators to ensure a thorough understanding of the study and the application of effective supervisory methods. A preliminary study was conducted on a group of ten older adults living in the specific study area to refine the data collection process. The data collection took place at community-based healthcare facilities, where elderly individuals were invited by local healthcare professionals. Following their health check-ups, participants were directed to a private room for the interview. Before proceeding, they were provided with a concise introduction to the research and asked to sign the informed consent documents. Each interview lasted between 30 to 40 minutes.

A structured survey was used to collect data, following a trial phase to ensure cultural validity, logical sequencing, and clarity of language. Input from healthcare workers and local elderly participants was integrated into the refinement of the survey before it was officially approved by the main researchers and local health facility leaders. The finalized survey contained five sections: demographic information, existing health conditions, and the EuroQol-5 Dimensions-5 Levels (EQ-5D-5L) for assessing HRQoL.

#### Demographic and anthropometric characteristics.

This section collected demographic and behavioral information, including age, gender, occupation, education level, marital status, living situation, body mass index (BMI), and blood pressure (systolic blood pressure [SBP] and diastolic blood pressure [DBP]). Additionally, participants provided self-reported data on alcohol consumption, tobacco use, and physical activity.

Blood pressure was specifically included due to its strong correlation with cardiovascular diseases, which are prevalent among elderly individuals and significantly impact HRQoL. While other physiological indicators such as blood sugar levels and mental health assessments were considered, they were recorded in the comorbidities section rather than in the demographic characteristics. This categorization was chosen because comorbidities, including diabetes and mental health disorders, required physician-confirmed diagnoses rather than self-reported or single-measurement data. Many participants presented with multiple comorbidities, necessitating a separate, detailed assessment beyond the scope of basic demographic profiling.

#### Comorbidities characteristics.

Physicians from the university and local health stations conducted clinical examinations and patient history reviews to assess and document the presence of comorbidities among participants. Diagnosed conditions were recorded in a medical record and categorized based on disease type, including hypertension, diabetes, osteoarthritis, cardiovascular diseases, chronic obstructive pulmonary disease (COPD), heart failure, and urinary diseases.

It is important to note that while commune health stations are capable of diagnosing and managing common chronic diseases, their diagnostic capabilities are generally limited for certain conditions that require specialized testing or evaluation by a specialist. For example, COPD, heart failure, and urinary diseases typically require diagnostic tests such as spirometry, echocardiography, and urinalysis, which are often unavailable at the commune health level. To address this limitation, physicians from the university provided sufficient medical equipment and expertise to conduct on-site screening and diagnosis for these conditions. This ensured that participants received a more comprehensive health assessment during the study. Additionally, participants with a history of chronic conditions were identified based on existing medical records, prior diagnoses from district or provincial hospitals, and physician-confirmed status during the study. For cases requiring further evaluation or specialized care beyond the capabilities of the research team, referrals were made to higher-tier hospitals where advanced diagnostic procedures could be conducted.

#### Health-related quality of life.

The participants’ HRQoL was evaluated using the EQ-5D-5L instrument, a widely recognized and validated international self-report tool for measuring health-related quality of life. This instrument covers five key dimensions: mobility, self-care, usual activities, pain/discomfort, and anxiety/depression. Each dimension is self-assessed across five response levels, which indicate the severity of the issue: no problem, slight problem, moderate problem, severe problem, or extreme problem [26]. The process involves generating a health state from every set of five responses, which can subsequently be transformed into a health utility through the utilization of a Vietnamese cross-walk value set [27]. The individuals who provided their response as the initial option were assigned to the “no problem” group, whereas any alternative responses were classified under the “having a problem” group. The EQ-5D-5L instrument is extensively employed within Vietnam, as evidenced by multiple sources [28–33], with the population norm =  0.91 (SD = 0.15) [28].

### Definitions of variables

Demographic variables included age, categorized into three groups (60–69, 70–79, and ≥ 80 years), and gender (male or female). Occupation was classified as either employed or retired/unemployed, while education level ranged from illiteracy to vocational training or higher. Marital status was categorized as single, having a spouse, separated/divorced, or widowed. Living arrangements were classified into three-generation, two-generation, one-generation families, living alone, or other.

Health behaviors were assessed through self-reported participation in physical exercise (yes/no), alcohol use (current drinker: yes/no), and smoking (current smoker: yes/no).

Anthropometric and clinical characteristics included body mass index (BMI), calculated as kg/m², and blood pressure, recorded as systolic (SBP) and diastolic (DBP) blood pressure in mmHg. A history of falling was determined based on whether the participant had experienced a fall in the past 12 months (yes/no). Comorbidities were categorized into neuropsychiatric, musculoskeletal, cardiovascular, respiratory, diabetes, and other diseases. The number of comorbidities was grouped into five categories: 0, 1–2, 3–4, 5–7, and ≥ 8.

Health-related quality of life (HRQoL) was assessed using the EQ-5D-5L instrument, measuring five dimensions: mobility, self-care, usual activities, pain/discomfort, and anxiety/depression. The EQ-5D-5L index score was calculated using the Vietnamese cross-walk value set to quantify overall health status.

### Statistical analysis.

The data analysis was conducted using Stata software version 16.0. The present study investigated variations in the EQ-5D index across diverse comorbidities. Multivariate Tobit regression, also known as censored regression, was used to explore the relationships between comorbidity and the EQ-5D index. The associations were subjected to adjustments in consideration of potential confounding variables. A forward stepwise selection strategy was used with a p-value of log-likelihood of < 0.2 to select variables into the model. A p-value of less than 0.05 was used to detect statistical significance.

## Results

Table 1 shows that among 3038 elderly people, most of them were female (61.0%) and aged from 60-69 years old (44.6%). The majority of participants were already employed (64.0%), had secondary education (44.4%) and lived with a spouse (82.0%). Living with a 3-generation family was the most common (50.8%). 16.6% of them had a fall in the last 12 months. The proportion of elderly people performing physical exercise, drinking alcohol and smoking tobacco was 66.1%, 21.4% and 18.3%, respectively.

**Table 1:** Demographic characteristics of participants.

Characteristics	Gender				Total	
	Male		Female			
	n	%	n	%	n	%
**Total**	1184	39.0	1854	61.0	3038	100.0
**Age group (years old)**						
60-69	550	46.5	805	43.4	1355	44.6
70-79	430	36.3	631	34.0	1061	34.9
≥ 80	204	17.2	418	22.6	622	20.5
**Occupation**						
Retired/Unemployed	419	35.4	675	36.4	1094	36.0
Employed	765	64.6	1179	63.6	1944	64.0
**Education**						
Illiteracy	11	0.9	22	1.2	33	1.1
Below primary education	85	7.2	172	9.3	257	8.5
Primary education	352	29.7	689	37.2	1041	34.3
Secondary education	568	48.0	781	42.1	1349	44.4
High school education	123	10.4	130	7.0	253	8.3
Vocational training or higher	45	3.8	60	3.2	105	3.5
**Marital status**						
Single	23	1.9	67	3.6	90	3.0
Have a spouse	1057	89.3	1434	77.4	2491	82.0
Separation/divorce	8	0.7	26	1.4	34	1.1
Widow	96	8.1	327	17.6	423	13.9
**Living arrangement**						
3-generation family	573	48.4	970	52.3	1543	50.8
2-generation family	348	29.4	486	26.2	834	27.5
1-generation family	243	20.5	333	18.0	576	19.0
Living alone	19	1.6	59	3.2	78	2.6
Others	1	0.1	6	0.3	7	0.2
**Have a history of falling**						
No	986	83.3	1548	83.5	2534	83.4
Yes	198	16.7	306	16.5	504	16.6
**Health Behaviors**						
Physical Exercise	776	65.5	1233	66.5	2009	66.1
Alcohol use	324	27.4	325	17.5	649	21.4
Smoking	270	22.8	287	15.5	557	18.3
	**Mean**	**SD**	**Mean**	**SD**	**Mean**	**SD**
**Age**	71.6	7.6	72.8	8.8	72.3	8.4
**Body mass index** (unit: kg/m^2^)	20.2	2.4	20.1	2.2	20.1	2.3
**Blood pressure**						
Systolic blood pressure (unit: mmHg)	131.9	15.7	131.8	15.8	131.9	15.8
Diastolic blood pressure (unit: mmHg)	80.4	7.6	80.3	7.7	80.4	7.7

Table 2 depicts patterns of comorbidities among the elder people. Only 9.5% of participants did not have any comorbidities. Cataracts were the most prevalent comorbidity (61.0%), followed by osteoarthritis (55.4%), rheumatoid arthritis (46.1%) and dementia (39.0%).

**Table 2:** Comorbidities patterns among participants.

Characteristics	n	%
**Neuropsychiatric disease**	1902	62.6
Stroke	260	8.6
Dementia	1186	39.0
Dizzy	861	28.3
Depression	990	32.6
Gait abnormalities	940	30.9
**Musculoskeletal diseases**	1684	55.4
Rheumatoid arthritis	1400	46.1
Osteoarthritis	1684	55.4
**Cardiovascular disease**	1130	37.2
Heart failure	96	3.2
Hypertension	1045	34.4
Postural hypotension	77	2.5
Vascular disease	28	0.9
**Respiratory disease**	116	3.8
COPD	44	1.5
Asthma	88	2.9
**Diabetes**	280	9.2
**Other diseases**	2053	67.6
Hearing disease	52	1.7
Cataracts	1853	61.0
Digestive diseases	489	16.1
Urinary disease	449	14.8
Dermatology	277	9.1
**Number of comorbidities**		
No comorbidity	289	9.5
1-2 comorbidities	813	26.8
3-4 comorbidities	812	26.7
5-7 comorbidities	739	24.3
≥ 8 comorbidities	385	12.7

**Fig 1:**
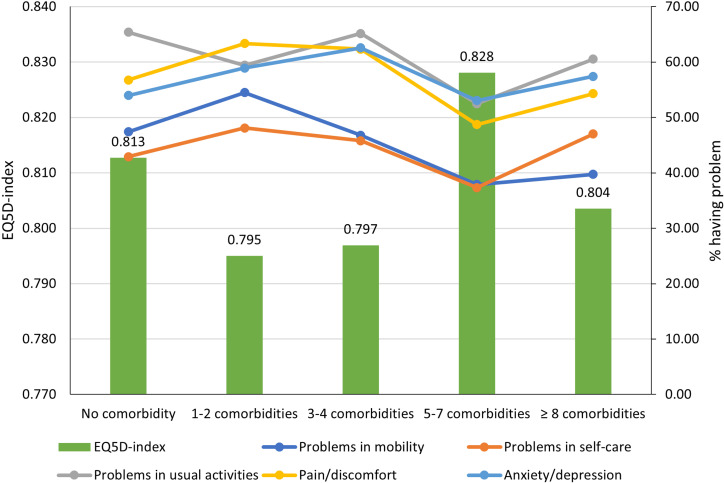
EQ-5D-5L index and dimensions in different groups regarding the number of comorbidities.

Fig 1 illustrates the distribution of participants encountering difficulties within each dimension as a function of the amount of comorbidities present. Overall, a significant majority of the participants, surpassing 30%, indicated experiencing difficulties across various dimensions. The mean EQ-5D index was 0.806 (SD = 0.184). Pain/discomfort, usual activities and anxiety/depression had the highest contributions regarding the reduction of the EQ-5D-5L index. Participants having 1-2 (mean = 0.795) or 3-4 comorbidities (mean = 0.797) had the lowest EQ-5D index compared to other groups.

**Fig 2:**
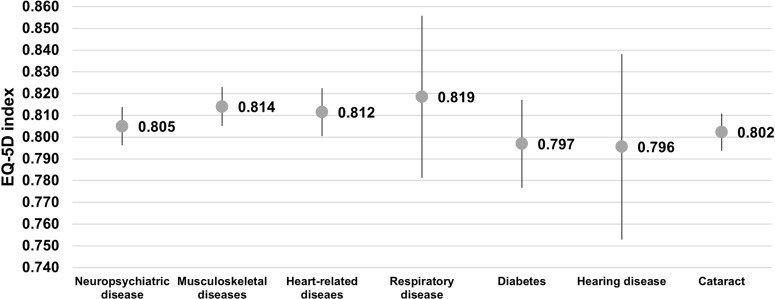
EQ-5D-5L index by different chronic conditions.

Fig 2 illustrates that participants having respiratory diseases had the highest EQ-5D index (mean = 0.819), followed by musculoskeletal diseases (mean = 0.814) and cardiovascular diseases (mean = 0.812). Participants with diabetes and hearing diseases had the lowest EQ-5D index (mean = 0.797 and 0796, respectively).

Table 3 shows that, after adjusting to other variables, participants with rheumatoid arthritis (β =  − 0.10; 95%CI =  − 0.13; − 0.07) and postural hypotension (β =  − 0.08; 95%CI =  − 0.14; − 0.02) had the highest EQ-5D index decrement, followed by urinary diseases (β =  -0.05, 95%CI = -0.09, -0.02) and Stroke (β =  -0.05, 95%CI = -0.09, -0.01).

**Table 3:** Associations between different comorbidities and EQ-5D index.

	EQ5D-index^¶^	
β	**95% CI**
**Neuropsychiatric Illness (Yes vs. No)**		
Stroke	-0.05***	-0.09; -0.01
Gait abnormalities	0.05***	0.03; 0.08
**Musculoskeletal Disease (Yes vs. No)**		
Rheumatoid arthritis	-0.10***	-0.13; -0.07
Osteoarthritis	0.12***	0.08; 0.15
**Cardiovascular disease (Yes vs. No)**		
Hypertension	0.03***	0.01; 0.05
Postural hypotension	-0.08***	-0.14; -0.02
**Respiratory Disease (Yes vs. No)**		
Asthma	0.05*	-0.00; 0.11
**Diabetes (Yes vs. No)**	-0.03**	-0.07; -0.00
**Other diseases (Yes vs. No)**		
Cataract	-0.02**	-0.04; -0.00
Digestive diseases	0.05***	0.02; 0.09
Urinary disease	-0.05***	-0.09; -0.02
Venereal diseases	0.04*	-0.00; 0.07

*** p < 0.01,

** p < 0.05,

* p < 0.1;

¶ adjusted for age, sex, education, marital status, occupation, living arrangement, and physical exercise.

## Discussion

This study elucidates the HRQoL among individuals aged 60 years or above living in rural communities of Vietnam. Overall, the EQ-5D index scores of elderly individuals were found to be lower in comparison to the broader population [28]. The findings emphasize the pronounced occurrence of comorbidities and a diminished HRQOL within this particular population. Additionally, a noteworthy decline in HRQOL is observed among individuals afflicted with specific comorbidities, particularly arthritis, postural hypotension, urinary diseases and stroke. The outcomes of this research have the potential to lay the groundwork for subsequent interventions aimed at enhancing HRQoL among general elderly individuals within the context of rural Vietnam.

In the present study, the prevalence of comorbidity was found to be more than 90%, which is substantially higher than a previous report among elder people with falls (75.6%) [25], as well as other studies in different regions [10,12,34,35]. The discrepancies observed in the prevalence rates across various studies can be attributed to variations in demographic characteristics, age composition, medical conditions, research methodology, and assessment tools employed. Cataracts were the most prevalent comorbidity (61.0%), followed by osteoarthritis (55.4%), rheumatoid arthritis (46.1%) and dementia (39.0%). Eyes and musculoskeletal issues, predominantly arthritis and osteoarthritis have previously been identified as commonly occurring among elderly people [25,23]. The high prevalence of comorbidities among elderly individuals in rural Vietnam is consistent with findings from other rural regions globally, such as China and India. Our study revealed that over 90% of participants had at least one comorbidity, with common conditions like cataracts, osteoarthritis, arthritis, and dementia. This aligns with the patterns seen in rural China, where multimorbidity rates among older adults are similarly high. Studies in China report rates as high as 90% for chronic multimorbidity, often involving complex combinations of cardiopulmonary, mental, and degenerative disorders, with hypertension frequently appearing as a comorbid condition[36,37]. The similarities between rural Vietnam and China suggest that elderly populations in low-resource rural settings across Asia are highly vulnerable to multimorbidity, significantly affecting their QoL. A comparable situation exists in rural India, where 79.7% of the elderly population has reported at least one chronic morbidity, with hypertension being the most common [38]. This further reinforces the findings in our study, where cardiovascular conditions, such as postural hypotension and stroke, were among the key contributors to reduced HRQoL.

When assessing HRQoL, this study demonstrates that pain/discomfort, usual activities, and anxiety/depression were the most significant contributors to the decline of HRQoL in elderly individuals residing in rural areas. This finding is consistent with previous studies on a cohort of elderly individuals who presented various health issues [39–42]. It is noteworthy that even among older individuals without any comorbidities, issues related to usual activities were reported most frequently, indicating that in older individuals living in rural areas, advanced age significantly affects their ability to perform daily activities and basic functions, thereby impacting their overall HRQoL. The mean EQ-5D-5L index score among elderly individuals in rural Vietnam was 0.806 (SD =  0.184), reflecting a moderate level of HRQoL This score is notably lower than the population norm for Vietnam, which is reported to be 0.91 (SD =  0.15) for the general population [43]. Access to healthcare, socioeconomic factors, and rural health challenges in Vietnam play a significant role in shaping these outcomes. Compared to urban areas, rural populations face distinct healthcare barriers, such as limited access to medical facilities, fewer healthcare providers, and financial constraints, which exacerbate health issues and contribute to lower HRQoL. These barriers are more pronounced in rural Vietnam compared to other countries like China, where rural healthcare infrastructure might be slightly better developed. A study conducted in Shanxi Province, China, found that rural older adults had a lower mean EQ-5D utility score (0.86 ±  0.23) compared to urban older adults (0.89 ±  0.22) [44], which is higher than what was observed in rural Vietnam. The socioeconomic context of rural Vietnam, marked by lower income levels and education, further complicates access to healthcare and influences the QoL outcomes. The findings from the multivariate regression model also indicate that the presence of certain comorbidities significantly influences the decline in HRQoL, including arthritis, postural hypotension, urinary diseases, and stroke. This finding is consistent with the aforementioned assertion, as well as with other related studies. [23,25]. Musculoskeletal dysfunctions have the potential to impede patients’ physical mobility, while heightened levels of dyspnea and fatigue may negatively impact the elder people’s HRQoL.

The outcomes of our study possess significant clinical implications. First, the phenomenon of gradual population ageing, coupled with enhanced life expectancy and the growing prevalence of comorbidity, serves to escalate the costs and intricacies associated with health resource allocation. The implementation of a robust healthcare system is imperative to guarantee its long-term viability and sustainable operation. Adopting HRQoL measures apart from the EQ-5D index could potentially facilitate a more comprehensive comprehension of the interplay between comorbidity and QoL. Second, given that more than 70% of the elder people with more than two comorbidities, it necessitates intricate care coordination and treatment. Hence, an imperative in the realm of healthcare is the formulation and implementation of clinical management strategies, alongside the establishment of specific clinical guidelines, to address the needs of individuals diagnosed with comorbid conditions. Such measures are essential, as they not only serve to decrease expenditures but also enhance the overall standard of care and health outcomes in this population. Finally, the findings of this study present an impetus for further investigation into the medical ailments that exert the most significant impact on the overall HRQoL of elderly people, specifically focusing on those coping with arthritis, postural hypotension, urinary diseases and stroke in advanced age.

The present study had several limitations in its design and implementation. Initially, an assessment of the severity and duration of every comorbidity was not conducted, thereby posing a potential influence on the participants’ HRQOL. Additionally, it should be noted that a cross-sectional design was employed within the confines of this study, thereby rendering it impossible to deduce causality between observed comorbidity and subsequent alterations in HRQOL. In addition, certain variables, such as nutritional status and muscle wasting were not assessed in the study. These factors have the potential to introduce confounding effects on the association between comorbidities and HRQOL. Hence, it should be warranted to initiate further longitudinal investigations to address this knowledge gap.

## Conclusion

This study showed a remarkably high prevalence of comorbidities among the elder people in rural communities of Vietnam. Additionally, arthritis, postural hypotension, urinary diseases and stroke were found to be associated with the highest reduction of EQ-5D index. Regular monitoring and screening of comorbidities play a crucial role in identifying individuals who would benefit the most from healthcare programs in improving HRQoL.
